# Diagnosis of infection utilizing accellix CD64

**DOI:** 10.1186/2197-425X-3-S1-A1013

**Published:** 2015-10-01

**Authors:** CL Sprung, RC Morales, H Kasdan, A Reiter, S Keren, J Meissonnier

**Affiliations:** Hadassah Hebrew University Medical Center, Department of Anesthesiology and Critical Care Medicine, Jerusalem, Israel; LeukoDx, Jerusalem, Israel

## Background/Purpose

Differentiating patients who are infected or not in the intensive care unit (ICU) can be very difficult. Present diagnostic tests remain inadequate. CD64 has been found to be a potentially useful marker to identify infected patients. Unfortunately, CD64 measured by standard flow cytometers in a laboratory takes hours to perform. The purpose of this study was to evaluate the Accellix CD64 instrument which provides results in 20 minutes in ICU patients with and without infections.

## Method

**Figure 1:**
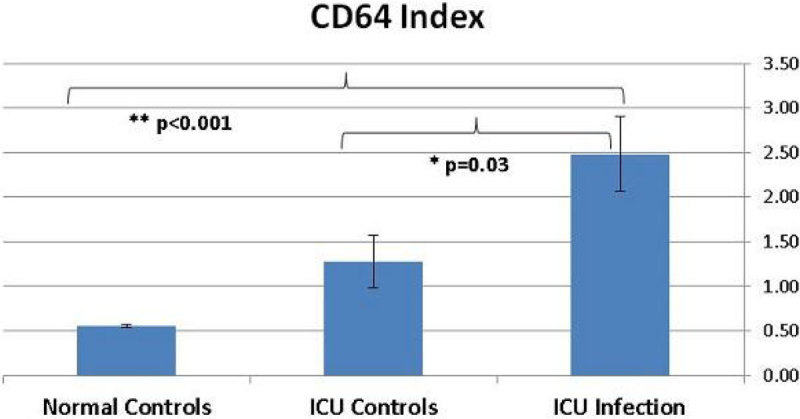
Figure 1

Infected (ICUi) and non-infected ICU patients (ICU Control-ICUc) and normal volunteers (C) had CD64 levels measured by the Accellix CD64 instrument. Measurements were calculated as 'CD64 index', i.e. the ratio between the fluorescence of the PMN population and the fluorescence of control beads. ICU infection, ICU control and normal control patients' results can be seen in Figure 1.

## Results

Sixty patients were studied (ICUi-17, ICUc-13 and C-30). CD64 Index levels were higher (mean ± SEM) in ICU infection patients then ICU control and normal control patients (2.49 ± 0.42 vs. 1.28 ± 0.3 vs. 0.56 ± 0.02. p = 0.03 for ICUi vs. ICUc, p < 0.001 for ICUi vs. C).

## Conclusion

CD64 Index levels are higher in infected than non-infected ICU patients. Accellix CD64 is a promising instrument to differentiate infected from non-infected ICU patients in a timely manner.

